# Efficacy of injection therapies in reducing hemiplegic shoulder pain: a systematic review and meta-analysis

**DOI:** 10.3389/fneur.2025.1634623

**Published:** 2025-09-30

**Authors:** Jing Nie, Hang Zhou, Shenao Du, Haowei Zhang, Yiying Liu, Xiangyang Wei, Wenhua Ning, Haiming Wang

**Affiliations:** ^1^Rehabilitation Medicine Department, The First Affiliated Hospital of Zhengzhou University, Zhengzhou, China; ^2^Center of Rehabilitation Engineering Technology Research, Henan Province, Zhengzhou, China

**Keywords:** injection therapies, hemiplegic shoulder pain, systematic review, meta-analysis, botulinum toxin

## Abstract

**Objective:**

This systematic review of randomized controlled trials (RCTs) with meta-analysis aimed to investigate the efficacy of injection therapies in reducing hemiplegic shoulder pain (HSP) in stroke survivors.

**Methods:**

PubMed, Embase and the Cochrane databases were searched from inception to April 20, 2025 to identify RCTs of stroke survivors with HSP undergoing injection therapies to reduce pain intensity. The main outcome of the assessment was the degree of pain relief as measured by visual analogue scale (VAS). And the secondary outcome indicator is the range of motion (ROM) at the end of the follow-up period.

**Results:**

A total of 408 results were identified by the search strategy, and 11 studies were included in the final analysis. We analyzed data for 353 stroke survivors with HSP, the results showed improvement of VAS within 4 weeks after injection was MD −1.03, 95% CI [−1.72, −0.33], *p* < 0.05, with large heterogeneity (I^2^ = 57%), and the improvement of VAS within 12 weeks after injection was MD −1.43 95% CI [−1.92, −0.94], *p* < 0.05, with no heterogeneity (I^2^ = 0%), significantly attenuated HSP. The improvement in shoulder external rotation ROM within 4 weeks after injection was MD 11.68, 95% CI [7.20, 16.15], *p* < 0.05, I^2^ = 0%, and the improvement within 12 weeks after injection was MD 10.00, 95% CI [5.78, 14.21], *p* < 0.05, I^2^ = 0%. The improvement in shoulder abduction ROM within 4 weeks after injection was MD 9.46, 95% CI [3.27, 15.64], *p* < 0.05, I^2^ = 0% while the improvement within 12 weeks after injection was MD 12.15, 95% CI [5.57, 18.73], *p* < 0.05, I^2^ = 7%.

**Conclusion:**

This systematic review and meta-analysis indicated that the addition of injection therapies to conventional rehabilitation is more effective than conventional rehabilitation alone in the complex treatment of patients with HSP in terms of both the short-term and long-term follow-up.

**Systematic review registration:**

The protocol for this systematic review and meta-analysis was prospectively registered with PROSPERO (CRD420251040988).

## 1 Introduction

Stroke is the second leading cause of death and disability worldwide, and post-stroke care and rehabilitation impose a significant economic burden at the individual and societal levels (1). From 1990 to 2019, the absolute number of stroke incidence has increased by 70%, the prevalence of stroke has increased by 85%, and the number of stroke deaths has increased by 43% (2). Hemiplegic shoulder pain is the most common complication in stroke patients, and it is diagnosed in anyone who experiences pain and discomfort in the affected shoulder at rest or during exercise after hemiplegia. Although estimates vary depending on study methods, the prevalence of HSP among stroke survivors is as high as 84%. Shoulder pain may appear early in the course of the disease, with a prevalence estimated at 17% in the first week and continuing to increase between 20 to 24% during recovery from 1 to 16 months after stroke (3).

The potential mechanisms of HSP include soft tissue pathology, impaired motor function, and CNS-related phenomena (4). Factors that may contribute to its appearance can be categorised as those related to the shoulder joint itself (rotator cuff injury or subluxation of the humeral head) (5) and those related to neurological disorders (lack of sensation, initial flaccid paralysis, hemispheric neglect and spasticity) (6). The persistence of HSP can lead to lifestyle disturbances as patients experience reduced range of motion, shoulder pain and subsequent upper limb dysfunction. HSP causes upper limb dysfunction in terms of motor function and dexterity, which can lead to difficulties in Activity of Daily Living Scale (ADL) (7). HSP can cause significant pain and reduced mobility, significantly impeding the rehabilitation process. High levels of pain often interfere with the patient’s rehabilitation process, so the main goal of HSP management is to reduce pain and increase shoulder ROM through an effective rehabilitation programme (8).

Injection therapies are a common treatment modality that can be used in all phases of shoulder pain in patients with hemiplegia and contribute to the patient’s recovery. And commonly used clinical drugs for shoulder nerve block therapy include botulinum toxin (BoNT), local anaesthetics, corticosteroid and hyaluronic acid (HA). Botulinum toxin has been widely used for the treatment of post-stroke spasticity, cervical dystonia, and muscular hyperactivity disorder. The mechanisms by which BoNT relieves pain include relaxation of overused muscles and inhibition of inflammatory injurious cytokines or neurotransmitters. Recently, BoNT injections have been increasingly used to treat musculoskeletal pain (9), and a study suggest it may have better analgesic properties (10). Steroids are more widely used in clinical practice due to their cheap price, low incidence of adverse effects without repeated use, and local anti-inflammatory effects. Precise intra-articular, intracapsular, peri-tendon attachment point, muscle trigger point or perineural injection therapy guided by X-ray, ultrasound, etc., can increase the local drug concentration and thus achieve the effective therapeutic goal (11). Steroids have a better short-term therapeutic effect, but long-term effectiveness needs to be further studied. Sodium hyaluronate has similar early effects to steroids. Sodium hyaluronate is an essential component of articular cartilage structure and function. Intra-articular sodium hyaluronate injections have the effect of reducing synovitis, regulating intra-articular osmotic pressure, protecting cartilage, preventing intra-articular adhesions, etc. They reduce the coefficient of friction of the joints, directly increase the viscosity and elasticity of synovial fluid, and provide cushioning for the joints (12).

Therefore, the aim of this systematic review and meta-analysis is to explore the clinical efficacy of injection therapies compared to conventional rehabilitation treatments.

## 2 Methods

This systematic review and meta-analysis was carried out with strict adherence to Preferred reporting of systematic review and meta-analysis (PRISMA) guidelines. Methodology of the study was pre-determined and delineated for smooth conduction of the review.

### 2.1 Research question

What is the clinical efficacy of injection therapies when compared to conventional rehabilitation treatment modalities in reducing pain and improving range of motion in patients with HSP?

### 2.2 PICO criteria

Population: Patients with hemiplegic shoulder pain.

Intervention: Treated with injection therapies, such as suprascapular nerve block, botulinum toxin and anesthetic.

Comparator(s)/control: Sham intervention, placebo, rehabilitation standard protocol.

Outcomes: Pain assessed with Visual Analogue Scale and Range of Motion.

### 2.3 Search strategy

We searched PubMed, Embase, Cochrane from the beginning to April 2025. The search string was developed using the following keywords: hemiplegic, stroke, shoulder, upper limb, pain, injection, anesthetic, botulinum toxin, corticosteroid, hyaluronic acid, randomized controlled trial, randomized. The systematic review protocol is available on the International Prospective Register of Systematic Reviews (PROSPERO; registration no.: CRD420251040988).

### 2.4 Study selection

Reports identified through various digital databases will be imported into Citation Manager (ENDNOTE) to eliminate duplication. Two reviewers will apply the eligibility criteria and select studies for inclusion in the systematic review, then two authors will independently screen the records for inclusion, with any disagreements between individual judgements being resolved by a third reviewer. Data will be extracted from the study files, including information on study design and methods. Both authors will extract and check the data received. For missing data, authors will be contacted for unreported data or other details.

### 2.5 Literature inclusion and exclusion criteria

#### 2.5.1 Inclusion criteria

1. Randomized clinical trials comparing the efficacy of injection therapies with conventional treatment for HSP. Case reports, reviews, animal experiments, retrospective studies, commentaries, or studies with incomplete data were also excluded.
2. Studies with an experimental group treated with injection therapies and control group treated with any other treatment modality like standard of care or placebo.
3. Studies reporting the efficacy in terms of alleviating pain or improvement in the range of motion.

#### 2.5.2 Exclusion criteria

1. Investigated shoulder pain on non-stroke patients.
2. Did not employ injection therapies to treat HSP.
3. Injected autologous blood-derived products (for the concern of significant variations in plasma components among different individuals).
4. Studies not reporting relevant outcomes.
5. Studies published in languages other than English.
6. Studies which are not randomized.

### 2.6 Data extraction

Data were extracted from the included reports by two independent reviewers and entered into an Excel spreadsheet. Data retrieved included: authors, year of publication, study design, nature of study and control groups, demographic characteristics (e.g., sample size, gender), injection therapies characteristics (e.g., type of injection therapies, site of injection), and reported outcomes (e.g., pain level and range of motion). The authors can be contacted by email if any information is missing or unclear.

### 2.7 Data analysis

The data were analysed both qualitatively and quantitatively. As part of the qualitative analysis, the demographic and intervention characteristics of the study were tabulated and summarised. Continuous results such as pain and range of motion are expressed as mean and standard deviation (SD). For studies that only provided median and interquartile spacing, we transformed the data according to the relevant methodology (13, 14) to overcome heterogeneity between study interventions and outcomes. All statistical analyses were performed with Review Manager 5.4.

Considering the heterogeneity among the included trials, using fixed effects model (I^2^ < 50%) or random effects model (I^2^ < 50%) to map the studies. Heterogeneity among the included trials was assessed using the I^2^ statistic. Values of I^2^ were categorized as either low (0 to <25%), moderate (25 to <50%), large (50 to <75%) or very large (≥75%).

### 2.8 Risk of bias analysis

Two independent reviewers analysed the risk of bias of included RCTs using the Cochrane Risk of Bias tool. Included trials were analysed for bias in selection of participants, bias in blinding of participants and staff, bias in blinding of outcome assessors, bias in selective reporting of outcomes and loss to follow-up by assessing the randomisation process and allocation concealment methods. Studies were categorised as low, medium or high risk based on the adequacy of the above aspects.

## 3 Results

### 3.1 Literature search and process results

A total of 408 results were identified by the search strategy, from PubMed (148), Embase (198), Cochrane (62). After deduplication, 133 studies were excluded, followed by 275 studies after preliminary screening.

**Figure 1:**
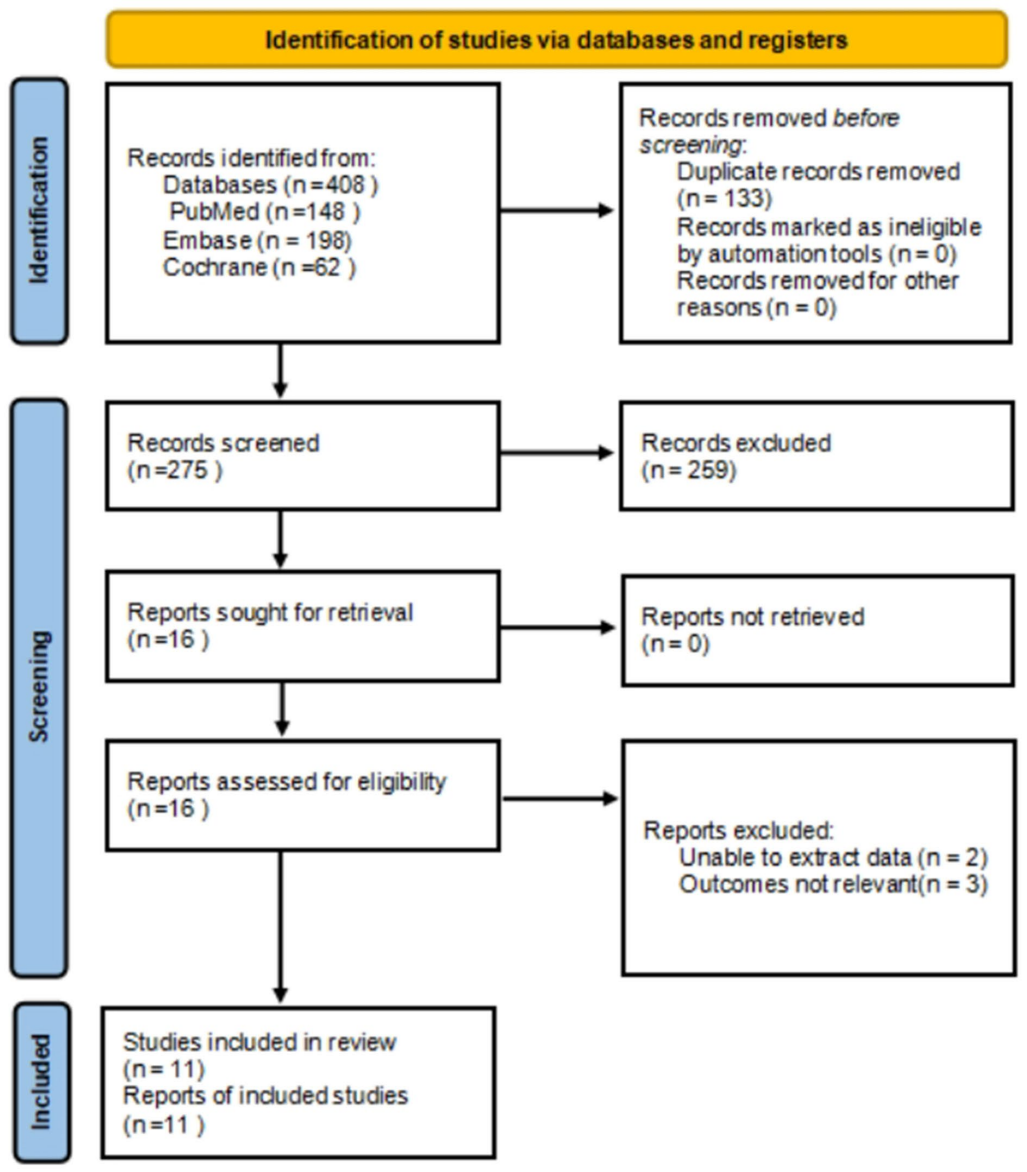
Study selection flow chart.

After a thorough screening of titles and abstracts, a total of 259 studies were deemed ineligible and thus excluded. Upon reviewing the full texts, a further 2 studies were excluded due to the inability to extract data. Additionally, 3 studies were excluded for irrelevance of their outcomes. Finally, 11 studies (15–25) were included in the final analysis (Figure 1).

The included studies were published from 2000 (21) to 2023 (15). Six of the RCTs compared botulinum toxin with placebo (15, 16, 20, 22–24), while one of the RCTs compared HA with placebo (17). And the last four articles compared corticosteroid with placebo (18, 19, 21, 25). A total of 353 patients (222 males and 131 females) were included in these 11 RCTs.

All included trials assessed pain relief after the intervention, and only nine trials (15–17, 19–24) assessed varying degrees of improvement in basic range of motion, such as abduction and external rotation. Demographic characteristics of the included studies and result are provided in Table 1.

**Table 1:** Characteristics of included studies.

Author	Year	Study design	Experimental		Control		Intervention	Comparison	Intervention protocol		Outcomes	Follow-up
			Sample size	Gender	Sample size	Gender			Dilution and dosage	Injection site		
De Melo Carvalho Rocha et al.	2023	RCT	12	8 M/4F	12	8 M/4F	BTA	placebo	diluted in 1 ml, 200 U	pectoralis major and subscapularis muscles	VAS; ROM (external rotation, abduction)	Baseline, 4 and12 weeks
Tan et al.	2021	RCT	18	15 M/3F	18	12 M/6F	BTA	placebo	reconstituted with 2.0 ml of saline at a concentration of 50 U/ ml, 100 U	subscapularis	VAS; ROM (external rotation, abduction)	Baseline, 1, 4, 12, and 24 weeks
Terlemez et al.	2020	RCT	10	7 M/3F	10	4 M/6F	SSNB (lidocaine + betamethasone)	lidocaine	5 ml 2% lidocaine + 1 ml betamethasone	suprascapular notch	VAS	Baseline, 1 and 4 weeks
Huang et al.	2018	RCT	18	11 M/7F	9	6 M/3F	sodium hyaluronate	placebo	2.5 ml sodium hyaluronate (ARTZ Dispo)	subdeltoid bursa	VAS; ROM (abduction)	Baseline, 4 and 12 weeks
Adey-Wakeling et al.	2013	RCT	32	21 M/11F	32	15 M/17F	SSNB (methylprednisolone + bupivacaine)	placebo	1 ml of 40 mg/ml methylprednisolone + 0.5% 10 ml bupivacaine	supraspinatus fossa	VAS	Baseline, 1, 4, and 12 weeks
Marciniak et al.	2012	RCT	10	6 M/4F	11	7 M/4F	BTA	placebo	Total 140 to 200 units BoNT (Botox) per person, with 100 to 150 units into pectoralis major muscles and 40 to 60 units into teres major muscles if shoulder extensors MAS ≥ 3	pectoralis major and teres major muscles	VAS; ROM (external rotation, abduction)	Baseline, 2, 4, and 12 weeks
Rah et al.	2012	RCT	29	21 M/8F	29	18 M/11F	triamcinolone acetonide + lidocaine	lidocaine	4 ml of 40 mg (10 mg/ml) triamcinolone acetonide + 1 ml of 1% lidocaine	subdeltoid bursa	VAS; ROM (external rotation, abduction)	Baseline, 2, 4, and 8 weeks
De Boer et al.	2008	RCT	10	6 M/4F	11	6 M/5F	BTA	placebo	50 units, dissolved in 1 ml of saline 0.9%	subscapularis muscle	VAS; ROM (external rotation)	Baseline, 6 and 12 weeks
Kong et al.	2007	RCT	7	3 M/4F	9	8 M/1F	BTA	placebo	500 units of BT-A were diluted with 2.5 ml of normal saline, and 250 units of BT-A were injected into the pectoralis major and biceps brachii, respectively, using anatomical landmarks	pectoralis major and biceps brachii muscles	VAS; ROM (abduction)	Baseline, 4, 8, and 12 weeks
Marco et al.	2007	RCT	14	10 M/4F	15	11 M/4F	BTA	placebo	500 units BoNT (Dysport)	pectoralis major muscles	VAS; ROM (external rotation, abduction)	Baseline, 1, 4, 12, and 24 weeks
Snels et al.	2000	RCT	18	12 M/6F	19	7 M/12F	triamcinolone acetonide	placebo	40 mg Kenacort A-40 in 1 ml; total 3 doses (0, 1st, 3rd week)	shoulder joints	VAS; ROM (external rotation)	Baseline, 3 weeks

### 3.2 Meta-analysis results

#### 3.2.1 Pain relief within 4 weeks after injection

**Figure 2:**
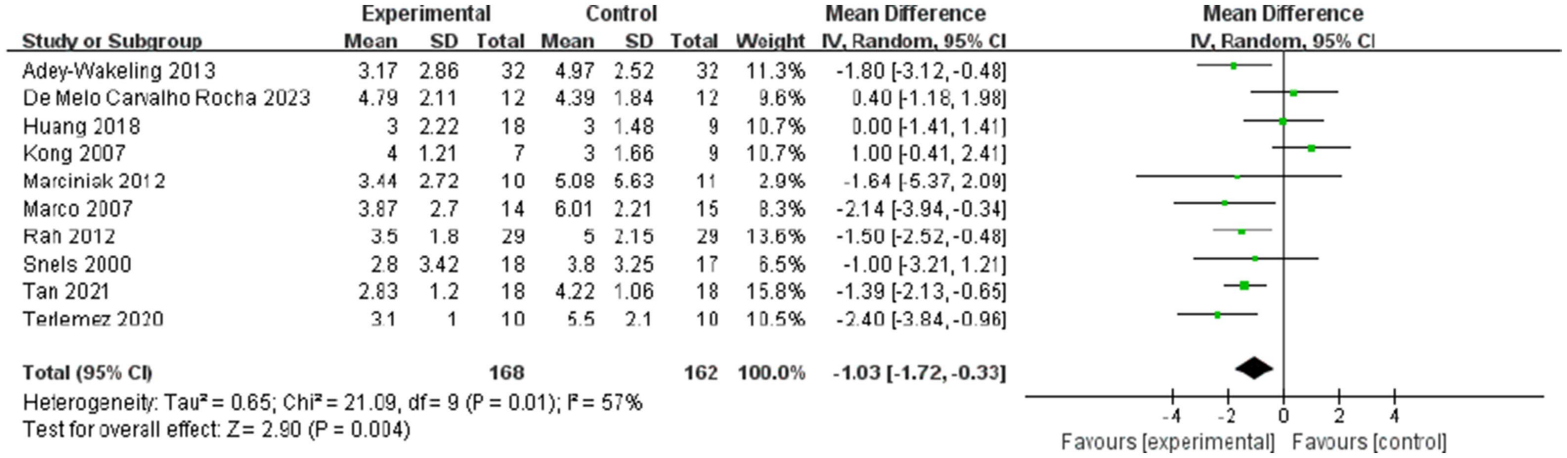
Forest plot showing comparison of pain relief within 4 weeks after injection between injection therapies and other treatment modalities.

The meta-analysis showed improvement of VAS within 4 weeks after injection was MD −1.03, 95% CI [−1.72, −0.33], *p* < 0.05, with large heterogeneity (I^2^ = 57%; Figure 2). The heterogeneity test showed low heterogeneity between studies, so a random effects model was used.

#### 3.2.2 Pain relief within 12 weeks after injection

**Figure 3:**
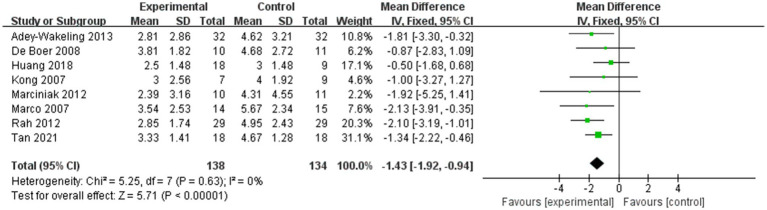
Forest plot showing comparison of pain relief within 12 weeks after injection between injection therapies and other treatment modalities.

The meta-analysis showed improvement of VAS at the end of treatment in the injection therapies group compared to the control group, with MD −1.43 95% CI [−1.92, −0.94], *p* < 0.05, with no heterogeneity (I^2^ = 0%; Figure 3). The heterogeneity test showed no heterogeneity between studies, so a fixed effects model was used.

#### 3.2.3 Shoulder external rotation in ROM

**Figure 4:**
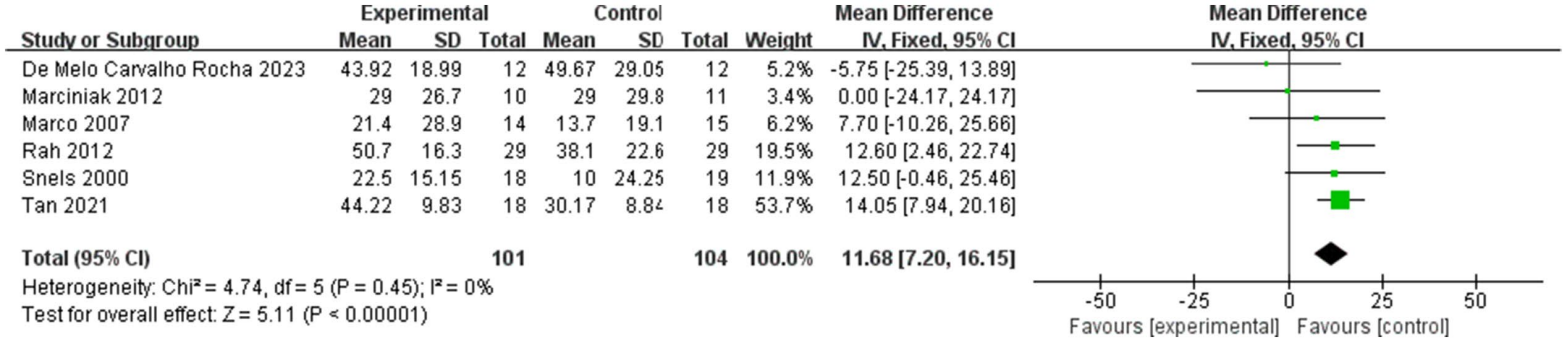
Forest plot showing comparison of improvement in ROM-external rotation within 4 weeks after injection between injection therapies and placebo.

**Figure 5:**
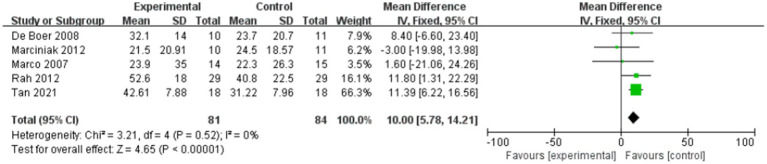
Forest plot showing comparison of improvement in ROM-external rotation within 12 weeks after injection between injection therapies and placebo.

Figure 4 shows the shoulder external rotation in ROM within 4 weeks after injection, while Figure 5 shows the shoulder external rotation in ROM within 12 weeks after injection.

Six studies assessed differences in the degree of improvement in shoulder external rotation ROM within 4 weeks after injection, with MD 11.68, 95% CI [7.20, 16.15], *p* < 0.05, with no heterogeneity (I^2^ = 0%; Figure 4). The heterogeneity test showed no heterogeneity between studies, so a fixed effects model was used.

And five studies assessed differences in the degree of improvement in shoulder external rotation ROM within 12 weeks after injection, with MD 10.00, 95% CI [5.78, 14.21], *p* < 0.05, with no heterogeneity (I^2^ = 0%; Figure 5). The heterogeneity test showed no heterogeneity between studies, so a fixed effects model was used.

#### 3.2.4 Shoulder abduction in ROM

**Figure 6:**
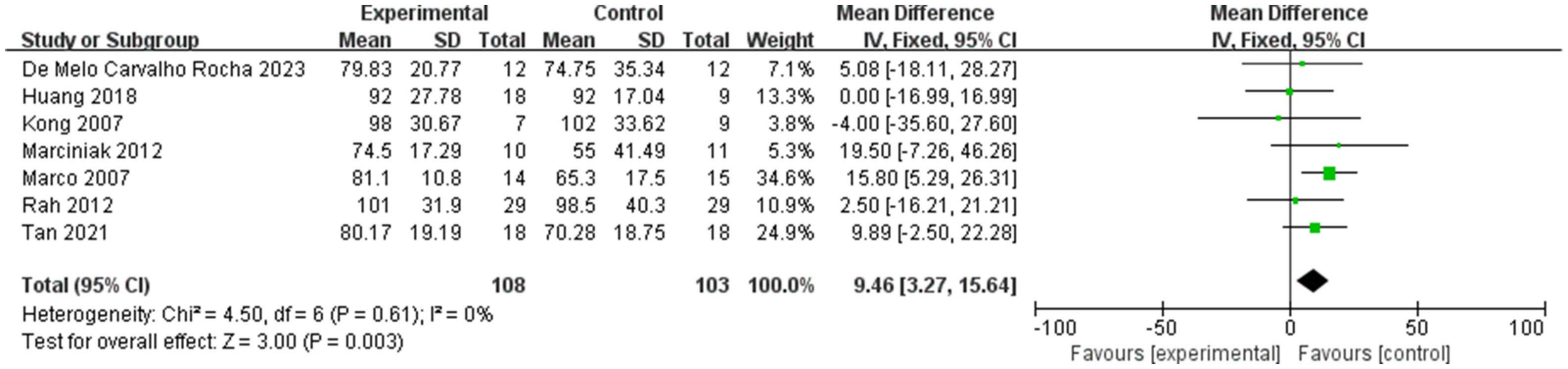
Forest plot showing comparison of improvement in ROM-abduction within 4 weeks after injection between injection therapies and placebo.

**Figure 7:**
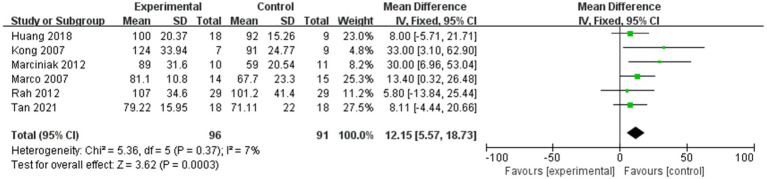
Forest plot showing comparison of improvement in ROM-abduction within 12 weeks after injection between injection therapies and placebo.

Figure 6 shows the shoulder abduction in ROM within 4 weeks after injection, while Figure 7 shows the shoulder abduction in ROM within 12 weeks after injection.

Seven studies assessed differences in the degree of improvement in shoulder abduction ROM within 4 weeks after injection, with MD 9.46, 95% CI [3.27, 15.64], *p* < 0.05, with no heterogeneity (I^2^ = 0%; Figure 6). The heterogeneity test showed no heterogeneity between studies, so a fixed effects model was used.

And six studies assessed differences in the degree of improvement in shoulder abduction ROM within 12 weeks after injection, with MD 12.15, 95% CI [5.57, 18.73], *p* < 0.05, with low heterogeneity (I^2^ = 7%; Figure 7). The low heterogeneity test showed low heterogeneity between studies, so a fixed effects model was used.

#### 3.2.5 Subgroup analyses for VAS

The 11 articles we included in the literature used different kinds of drugs for injection therapies. Five of the RCTs compared botulinum toxin with placebo, while one of the RCTs compared hyaluronic acid with placebo. And the last four articles compared corticosteroid with placebo. In order to further compare the efficacy of different types of drug injections for HSP, subgroup analyses were done in the botulinum toxin group and the corticosteroid group.

**Figure 8:**
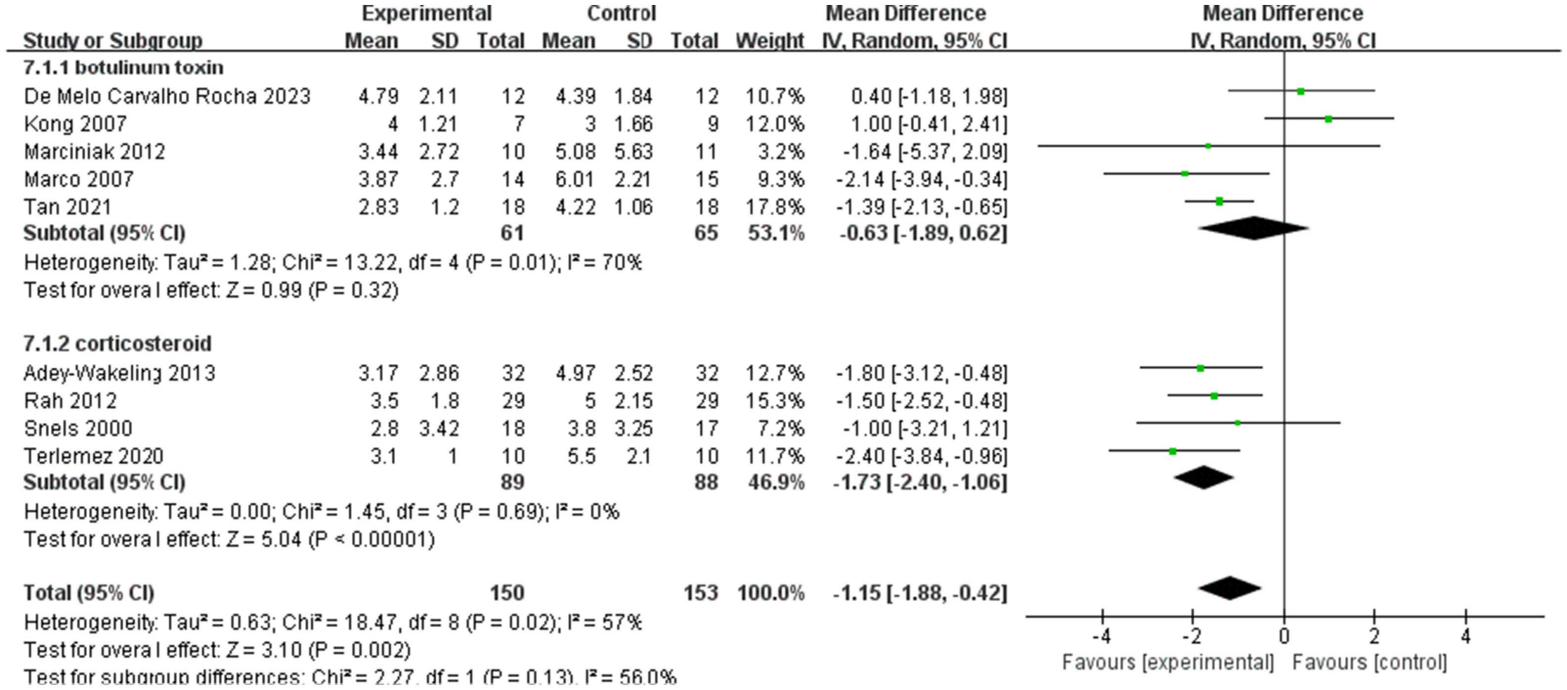
Subgroup analyses of the improvement of VAS within 4 weeks after injection.

**Figure 9:**
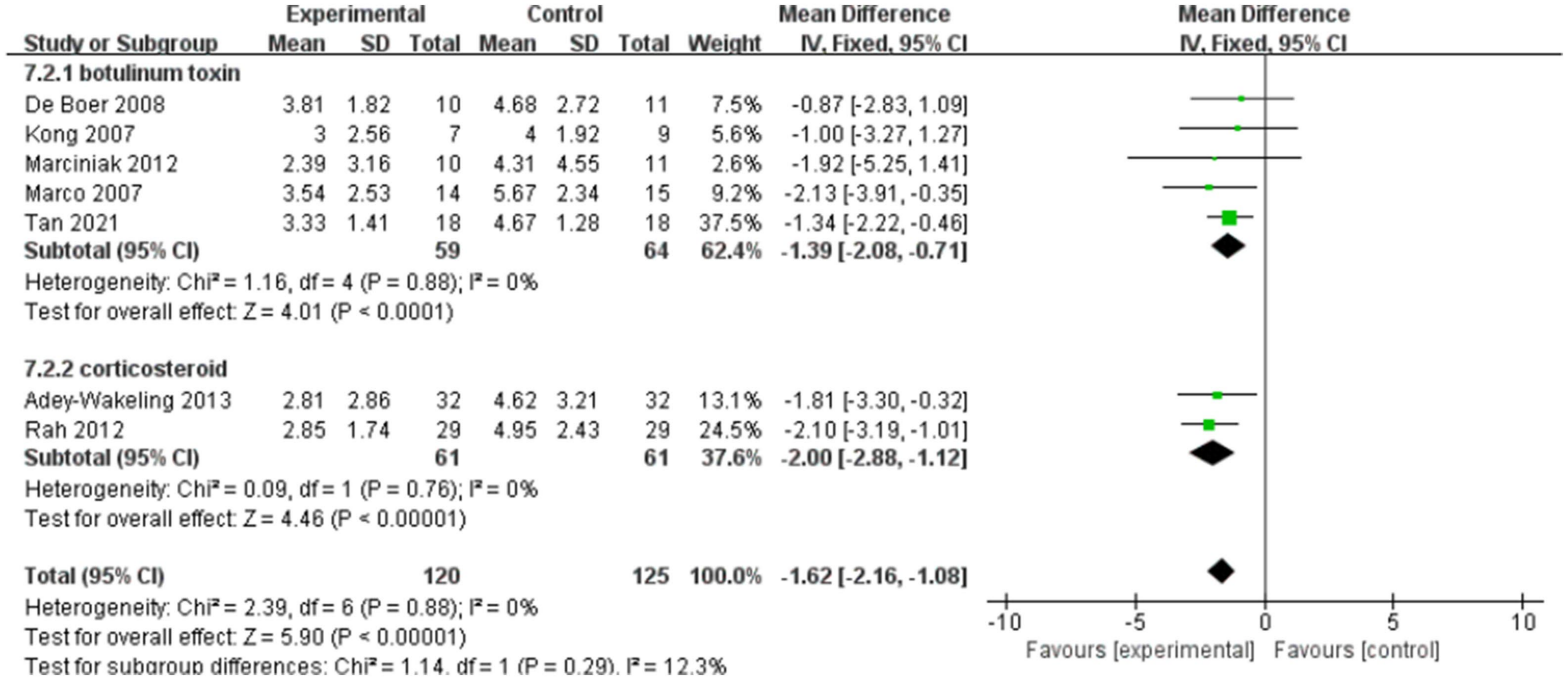
Subgroup analyses of the improvement of VAS within 12 weeks after injection.

Figure 8 shows the subgroup analyses for VAS within 4 weeks after injection, while Figure 9 shows the subgroup analyses for VAS within 12 weeks after injection.

Subgroup analyses within 4 weeks of injection showed MD −1.15, 95% CI [−1.88, −0.42], *p* < 0.05, with large heterogeneity (I^2^ = 57%). In addition, MD of the botulinum toxin group is −0.63, with 95% CI [−1.89, −0.62], *p* > 0.05, with large heterogeneity (I^2^ = 70%). In the corticosteroid group, the value of MD is −1.73, with 95% CI [−2.40, −1.06], *p* < 0.05, with no heterogeneity (I^2^ = 0%; Figure 8).

Subgroup analyses within 12 weeks of injection showed that MD of the botulinum toxin group is −1.39, with 95% CI [−2.08, −0.71], *p* < 0.05, with no heterogeneity (I^2^ = 0%). In the corticosteroid group, the value of MD is −2, with 95% CI [−2.88, −1.12], *p* < 0.05, with no heterogeneity (I^2^ = 0%). And the total heterogeneity of subgroup analyses is 0% (Figure 9).

### 3.3 Risk of bias assessment

**Figure 10:**
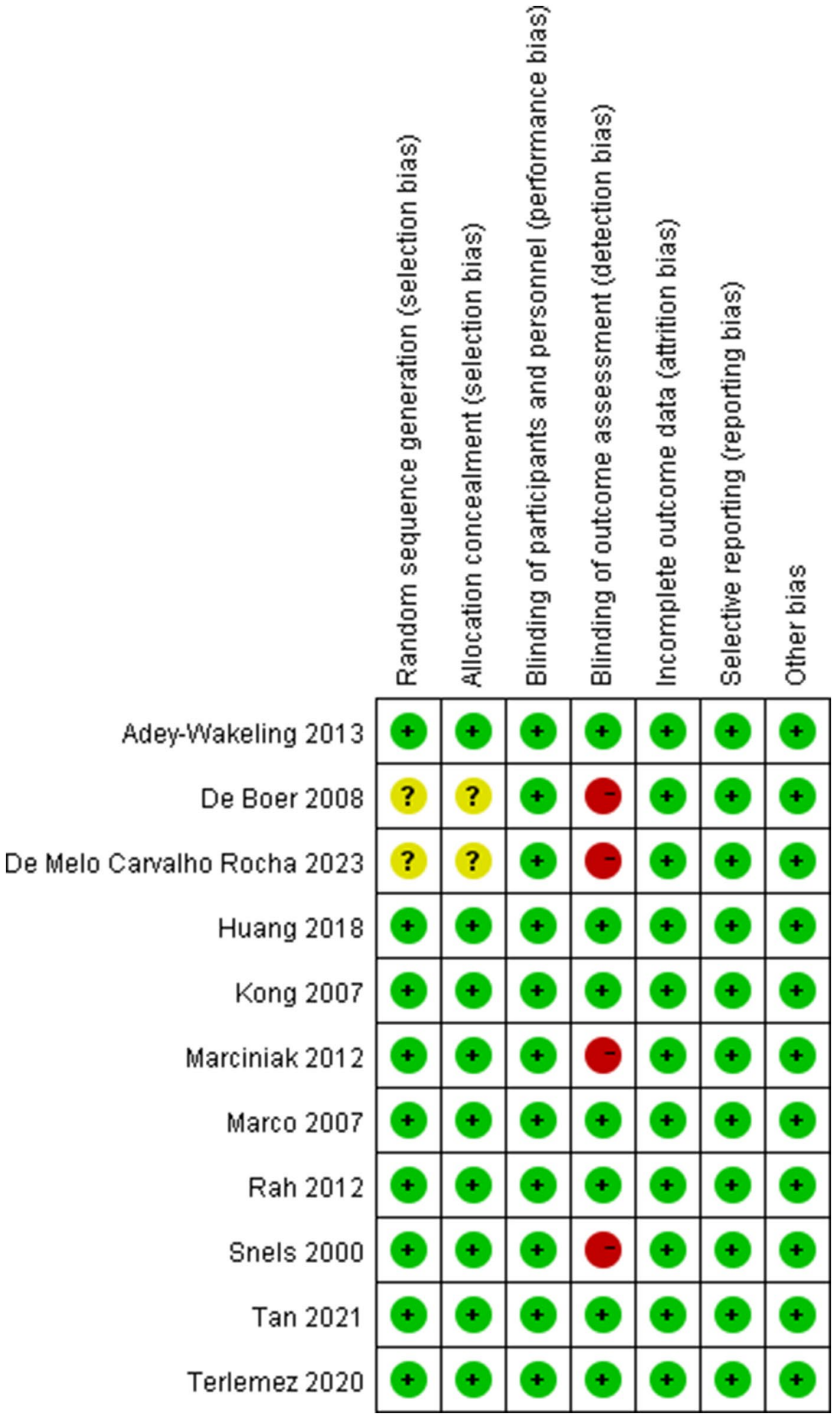
Risk of bias assessment summary of all included trials.

**Figure 11:**
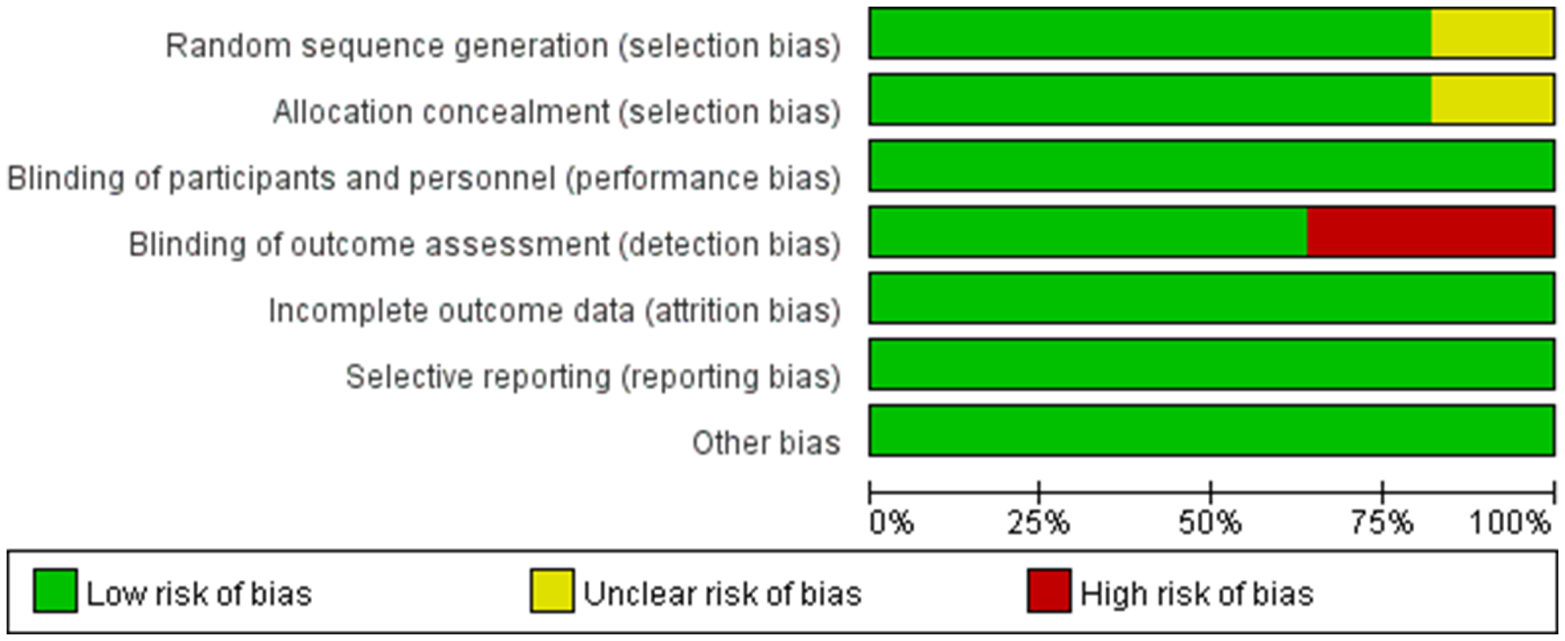
Risk of bias assessment summary of all included trials.

The quality of included trials was medium to high. Five studies were low risk in all aspects of assessed risk. Two studies did not provide selection bias, for which the respective domains were marked at unclear risk. And four studies were not blinded to the outcome assessment, for which the respective domains were marked at high risk (Figures 10, 11).

## 4 Discussion

This systematic review and meta-analysis compared the efficacy of injection therapies with other treatments for HSP, and the outcomes assessed included VAS scales, shoulder external rotation ROM and shoulder abduction ROM.

Among the available studies, a study by de Sire et al. (26) demonstrated that botulinum toxin type A injections, suprascapular nerve pulsed radiofrequency, suprascapular nerve blocks, and trigger-point dry needling significantly reduced HSP compared with conventional rehabilitation, demonstrating the superiority of rehabilitation techniques. A network Meta-Analysis by Chiu et al. (27) showed that all five injection therapies, suprascapular nerve block, IMBoNT, IBBoNT, Steroid, and HA, were more effective than placebo in reducing HSP. At week 4 post-intervention, SSNB had the best efficacy, followed by intramuscular BoNT injection. Between weeks 4 and 24, intramuscular BoNT injections appeared to be the most effective alternative for the treatment of HSP.

Our study selected randomized controlled trials and focused on the efficacy of injectable therapies for HSP in terms of both short-term efficacy and long-term efficacy. Our study showed that, pain relief at 4 weeks post-injection was MD −1.03, 95% CI [−1.72, −0.33], *p* < 0.05, I^2^ = 57%, and pain relief at 12 weeks post-injection also suggests that injection therapies improve pain in HSP patients compared to controls (I^2^ = 0%, 95% CI [−1.92, −0.94], *p* < 0.05). Improvement in the ROM of shoulder external rotation was observed at 4 weeks post-injection (I^2^ = 0%, 95% CI [7.20, 16.15], *p* < 0.05) and at 12 weeks post-injection (I^2^ = 0%, 95% CI [5.78, 14.21], *p* < 0.05) compared to the control group. Meanwhile, shoulder abduction in ROM at 4 weeks post-injection was statistically significant (I^2^ = 0%, 95% CI [3.27, 15.64], *p* < 0.05), and shoulder abduction in ROM at 12 weeks post-injection also suggests that injection therapies improve the ROM of shoulder abduction in HSP patients compared to controls (I^2^ = 7%, 95% CI [5.57, 18.73], *p* < 0.05). The results shows that the addition of injection therapies to conventional rehabilitation is more effective than conventional rehabilitation alone in the complex treatment of patients with HSP in terms of both the short-term and long-term follow-up. This is also relevant to our clinical work, reflecting the stability of the effectiveness of injection therapies for HSP.

In addition, we performed subgroup analyses of different injection therapy types. Subgroup analyses of the improvement of VAS within 4 weeks after injection showed that the total heterogeneity of subgroup analyses is 57%, while the value of I^2^ is 70% in botulinum toxin group (*p* > 0.05) and 0% in the corticosteroid group (*p* < 0.05). Subgroup analyses of the improvement of VAS within 12 weeks after injection showed that the total heterogeneity of subgroup analyses is 0%, while the value of I^2^ is 0% in both botulinum toxin group and the corticosteroid group (*p* < 0.05). The results of the subgroup analyses showed that the botulinum toxin group did not have a significant effect in the short-term period of 4 weeks post-injection, but both botulinum toxin and corticosteroids were significantly effective against HSP in terms of long-term efficacy. It is worth noting that there are some differences between our study and existing studies regarding the differences in short- and long-term efficacy of botulinum toxin for HSP. A study by Xie et al. (10) revealed a statistically significant decrease in the VAS score in the BTX group vs. the control group at 1, 4, and 12 weeks post injection. Another meta-analysis by Li et al. (28) showed that BTA significantly reduced pain at 1 week (SMD = −0.93; 95% CI [−1.67, −0.19]; *p* = 0.01) and 4 weeks (SMD = −0.90; 95% CI [−1.51, −0.28]; *p* < 0.01), but not at 12 weeks compared to placebo. The reason for the difference in the results of the meta-analysis may be the difference in the inclusion criteria and the selection of the database; we studied the effect of injectable therapy on HSP, so the control group was selected only for placebo or conventional rehabilitation. Other studies included a comparison of botulinum toxin and nerve blocks, with no language restrictions applied.

**Figure 12:**
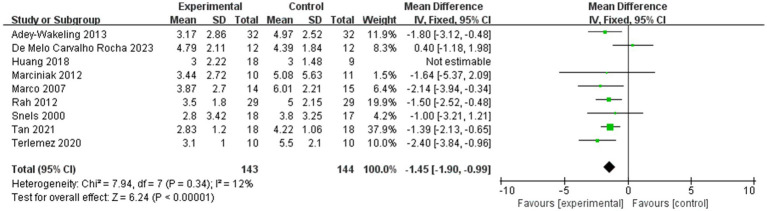
Sensitivity analysis of the improvement of VAS within 4 weeks after injection.

Next, we conducted a sensitivity analysis, and after excluding the literature one by one, we found that after deleting the article of Kong et al. (23), the results showed that MD of VAS for the sensitivity analysis is −1.45, with 95% CI [−1.90, −0.99], *p* < 0.05, with low heterogeneity (I^2^ = 12%; Figure 12). This suggests that this literature may be a source of heterogeneity. Upon further analysis, in the article by Kong et al., we found that the pectoralis major and biceps brachii muscles were selected as injection sites, which was different from other studies. The difference in inclusion criteria may have contributed to the heterogeneity.

**Figure 13:**
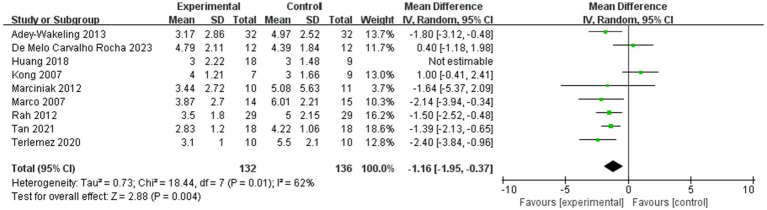
Sensitivity analysis of the improvement of VAS within 4 weeks after injection.

It is important to note that in our study, we selected 4 weeks and 12 weeks post-injection as the time points for outcome measures. Most existing studies use 4 weeks and 12 weeks as follow-up time points. As is well known, BTX-A injection typically begins to take effect approximately 1 week post-injection, reaches peak efficacy at 4 weeks, and maintains efficacy for 3 to 6 months (29). Since nerve endings regenerate within 8 to 12 weeks post-injection, the observed efficacy also decreases over time. Therefore, we defined 4 weeks post-injection as the short-term period and 12 weeks post-injection as the long-term period. Our results represent the average effect within the corresponding time window, and differences in short-term and long-term follow-up time points across studies may be a source of heterogeneity in the literature. After excluding the literature that selected follow-up results at 3 weeks as short-term efficacy, the direction of the combined results did not change and the results remained significant (*p* < 0.05; Figure 13), suggesting that the results of the meta-analysis were stable.

**Figure 14:**
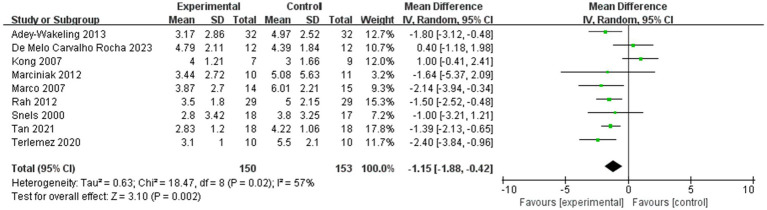
Sensitivity analysis of the improvement of VAS within 4 weeks after injection.

**Figure 15:**
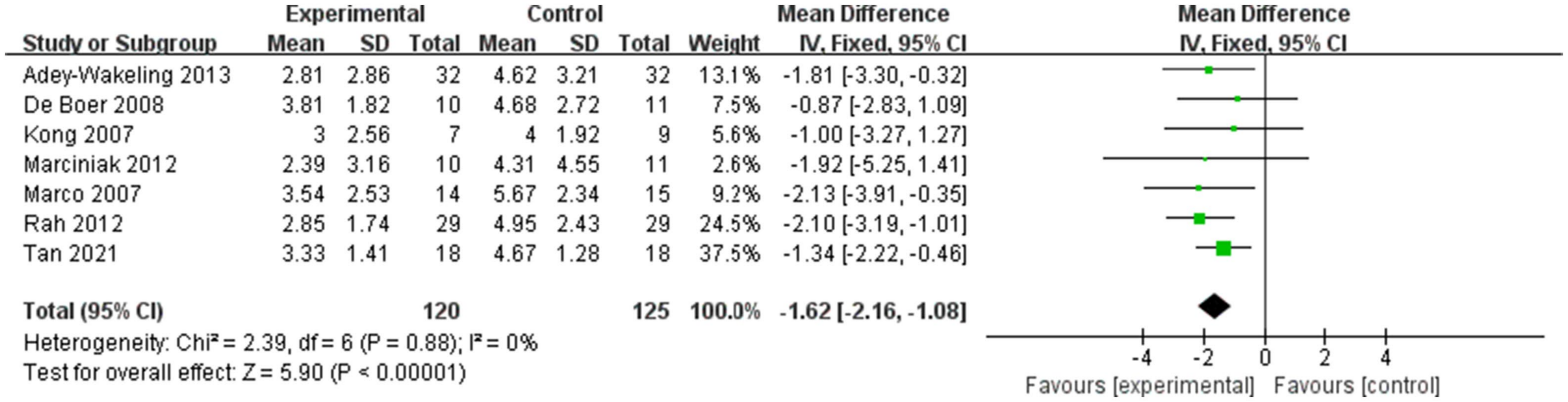
Sensitivity analysis of the improvement of VAS within 12 weeks after injection.

It should also be noted that there was only one article in the hyaluronic acid group in our study, so the hyaluronic acid group was not examined in the subgroup analyses. Therefore, we performed further sensitivity analyses (Figures 14, 15). After excluding the literature from the hyaluronic acid group (17), the direction of the combined results did not change and the results remained significant (*p* < 0.05), suggesting that the results of the meta-analysis were stable. Unfortunately, there is not much literature available regarding the use of hyaluronic acid injection for the treatment of HSP. And the article we included on the injection of hyaluronic acid has a high quality evaluation. This suggests that in future clinical studies, clinical practitioners can further explore the specific effects of hyaluronic acid injections on HSP patients, thereby bringing more possibilities for the treatment of HSP.

In our study, we discussed the efficacy of three injection therapies for shoulder pain: botulinum toxin 、corticosteroids, and hyaluronic acid. Additionally, there have been some innovative and highly promising new explorations in the treatment of painful musculoskeletal disorders. A research by Vascellari et al. (30) indicated that innovative bio-orthopaedic methods, particularly platelet-rich plasma (PRP) and mesenchymal stem cells (MSCs), can shorten recovery time for muscle injuries and reduce the risk of re-injury by regulating inflammation and promoting tissue regeneration. Future studies require higher-quality design, implementation, and reporting to investigate whether MSCs and PRP may serve as an innovative conservative treatment strategy for HSP.

In conclusion, injection therapies were found to significantly reduce shoulder pain and improve the ROM in external rotation and abduction in patients with HSP compared to the control group in both short-term and long-term follow-up. These results are consistent with those reported in previous studies.

The present systematic review has some limitations. Firstly, we did not further compare differences in outcome metrics for longer follow-ups. Second, our inclusion criteria median selected clinical randomised controlled trials with VAS or NRS as indicators of pain reduction. Future studies with larger sample sizes and longer follow-up times are needed to provide more robust evidence.

## 5 Conclusion

This systematic review and meta-analysis suggests that the addition of injection therapies to conventional rehabilitation is more effective than conventional rehabilitation alone in the complex treatment of patients with HSP in terms of both the short-term and long-term follow-up.

## Data Availability

All data generated or analysed in this study are included in this published article.
